# Mechanisms Underlying Altitude-Induced and Group 3 Pulmonary Hypertension

**DOI:** 10.3390/ijms27020572

**Published:** 2026-01-06

**Authors:** Giuseppina Milano, Sara Ottolenghi, Gustavo Zubieta-Calleja, Maurice Beghetti, Michele Samaja

**Affiliations:** 1 Department Coeur-Vaisseaux, Cardiac Surgery Center, University Hospital of Lausanne, 1005 Lausanne, Switzerland; giuseppina.milano@chuv.ch; 2 Department of Medicine and Surgery, University of Milano Bicocca, 20126 Milan, Italy; s.ottolenghi@campus.unimib.it; 3 Department of Emergency, Ospedale San Carlo Borromeo, ASST Santi Paolo e Carlo, 20146 Milan, Italy; 4 High Altitude Pulmonary and Pathology Institute (HAPPI-IPPA), La Paz 0201, Bolivia; zubieta@altitudeclinic.com; 5 Unité de Cardiologie Pédiatrique, University Hospital of Geneva, University of Geneva, 1205 Geneva, Switzerland; maurice.beghetti@hug.ch; 6 Department of Health Science, University of Milan, 20122 Milano, Italy

**Keywords:** Group 3 pulmonary hypertension, chronic hypoxia, hypoxic pulmonary vasoconstriction, vascular remodeling, redox imbalance, PI3K–Akt signaling, Na^+^/H^+^ exchange, nitric oxide, autophagy, mitochondrial dynamics, mitophagy, metabolic reprogramming, erythropoietin signaling, inflammation

## Abstract

Pulmonary hypertension is a progressive and life-threatening disorder affecting approximately 1% of the global population, with increasing prevalence among elderly individuals. Although it most commonly arises as a complication of chronic cardiac or pulmonary diseases, it may also develop in otherwise healthy individuals exposed to chronic hypoxia at high altitude. In this setting, sustained alveolar hypoxia triggers pulmonary vasoconstriction and vascular remodeling, key processes driving the elevation of pulmonary arterial pressure and highlighting the critical role of environmental stressors in disease pathogenesis. In this review, we examine the molecular mechanisms underlying the hypoxia-pulmonary hypertension axis, focusing on the complex and interconnected signaling networks involving redox imbalance, PI3K–Akt signaling, Na^+^/H^+^ exchange, nitric oxide bioavailability, autophagy, mitochondrial dynamics and mitophagy, metabolic reprogramming, inflammation, adventitial remodeling with particular emphasis on pulmonary arterial adventitial fibroblasts, and erythropoietin signaling. We also discuss current knowledge gaps and emerging therapeutic opportunities that may arise from a deeper understanding of these pathways. Collectively, while many of the signaling mechanisms implicated in hypoxia-induced pulmonary hypertension offer therapeutic promise, none have yet proven fully translatable, underscoring the multifactorial and tightly integrated nature of this disease.

## 1. Introduction

Pulmonary hypertension (PH) is a lung vascular disorder that affects approximately 1% of the global population, with prevalence rising to 10% among individuals over the age of 65 [1]. PH is defined by a mean pulmonary arterial pressure (mPAP) > 20 mmHg at rest. This may derive from the interplay of several pathological mechanisms that include proliferation of pulmonary arterial smooth muscle cells (PASMC) and endothelial cells (PAEC), infiltration of inflammatory cells, muscularization of lung arterioles, and thickening of the medial and intimal layers of pulmonary arteries [2]. Increased mPAP contributes to progressive obstruction of the pulmonary vasculature, right ventricular (RV) hypertrophy and failure. Despite important progress and advances in treatment, PH is still associated with high morbidity and mortality. Therefore, a deeper understanding of the underlying molecular mechanisms is needed to develop novel and more effective therapeutic strategies.

Increased mPAP develops secondary to several pathologies. In the most recent World Symposium, they have been subdivided into five categories [3], subsequently refined for adult and pediatric contexts (Table 1). Group 3 PH patients are a heterogeneous group that includes diseases secondary to hypoxia or hypoventilation, as well as developmental diseases typical of the pediatric contexts. This heterogeneity should be taken into consideration when discussing the processes leading to PH in Group 3, but in this manuscript the focus is on hypoxia-related PH.

A distinct form of PH associated with chronically reduced arterial O_2_ tension, altitude-induced PH (AIPH), is commonly observed in otherwise healthy individuals residing at high altitude. This condition primarily arises from hypoxic pulmonary vasoconstriction (HPV), a physiological mechanism that redirects blood flow away from poorly oxygenated alveoli to optimize ventilation–perfusion matching. Whether HPV represents an adaptive or maladaptive process remains uncertain, but it is likely that when the initial adaptive vasoconstriction becomes chronic, the ensuing persistent HPV contributes to progressive structural remodeling of the pulmonary vasculature. Hypoxia alone cannot account for pathological PH, as this would imply that all high-altitude residents experience the disease, which is not the case as extended longevity has been documented in these populations [4]. Nonetheless, prolonged life at high altitude may accelerate the aging process, as supported by studies in populations living at very high elevations (>3500 m), whereas accumulating evidence suggests that residence at moderate altitude (1500–2500 m) confers protective effects on several physiological systems [5]. The vascular remodeling observed in AIPH closely parallels that of Group 3 PH, encompassing smooth muscle cell proliferation, intimal thickening, adventitial fibrosis, progressive increases in mPAP, RV hypertrophy, and, in severe cases, RV failure.

Regional studies highlight that AIPH is a heterogeneous condition influenced by diverse genetic, environmental, dietary, and metabolic factors. AIPH presents some variability across highland populations, driven by distinct evolutionary and physiological adaptive strategies. Peruvian and Bolivian highlander populations display high resting pulmonary pressures and prominent pulmonary vascular remodeling, with a prevalence of 5–18% at elevations above 3000 m [6]. However, a recent metanalysis showed that chronic mountain sickness (CMS) might be generated not from hypoxemia but by multiple associated diseases [7]. Usually, mildly elevated mPAP may occur during exercise, typically 29–33 mmHg (95% CI), but mPAP is within normal limits, typically 16–20 mmHg (95% CI) at rest [8,9]. Central Asian (Kyrgyz) highlanders exhibit high (14–20%) AIPH prevalence, linked to peculiar angiotensin-converting enzyme genotypes and vascular responses [10]. By contrast, Tibetan highlanders show a distinct adaptation phenotype, marked by comparatively low hemoglobin (Hb) levels and reduced HPV, features that apparently lead to a very low occurrence of CMS and AIPH. Numerous studies confirm that Tibetans have significantly lower mPAP than Andeans at similar altitudes [11,12,13]. Another adaptive pattern was observed in Ethiopian (Amhara) highlanders, who maintain near sea-level Hb levels despite living at 3000–3700 m, but often display mildly elevated mPAP without increased pulmonary vascular resistance or symptomatic CMS, which indicates a hemodynamic profile fundamentally different from both Andeans and Tibetans [14]. It is important to stress, however, that dietary differences, such as higher iron intake in the Andeans relative to Tibetans, can be misleading, because of its effects on red blood cell production, which when increased beyond certain limits augments blood viscosity, thereby influencing PH onset [9].

This review aims to elucidate the hypoxia–PH axis, highlighting how the cardiopulmonary response to hypoxia contributes to PH development and progression. We examine the key molecular pathways activated by hypoxia that underlie PH pathogenesis, with emphasis on the dual role of hypoxia—as a primary driver of PH in otherwise healthy individuals exposed to high altitude, and as a secondary consequence of chronic pulmonary diseases (Group 3 PH), where alveolar hypoxia exacerbates vascular remodeling and sustains a self-perpetuating cycle of disease progression.

We regret the inadvertent omission of some relevant references. In preparing this narrative review, we deliberately prioritized seminal and conceptually foundational studies over subsequent confirmatory reports, with the aim of acknowledging the original descriptions and initial mechanistic insights underlying the phenomena discussed. Evidence derived from animal models was included primarily in instances where corresponding data from human studies were limited, inconclusive, or unavailable. This approach was intended to emphasize conceptual development while maintaining translational relevance.

## 2. Hypoxia Sensing

Hypoxia is defined as any situation whereby the O_2_ supply is insufficient to match the body’s needs. It may arise either from reduced O_2_ availability in the environment (e.g., altitude hypoxia) or from any restriction along the O_2_ cascade from atmosphere to mitochondria [15]. In the case of pulmonary diseases, the barrier to the O_2_ flow from air to the mitochondria may arise either at the atmosphere-to-alveoli or at the alveoli-to-arterial blood interface. Whichever the cause, the response of the organism to hypoxia, and consequently the activation of adaptive processes, necessarily involves the O_2_ sensing machinery, e.g., the molecular systems that transduce the O_2_ lack into a signal.

### 2.1. Transducers

**Figure 1:**
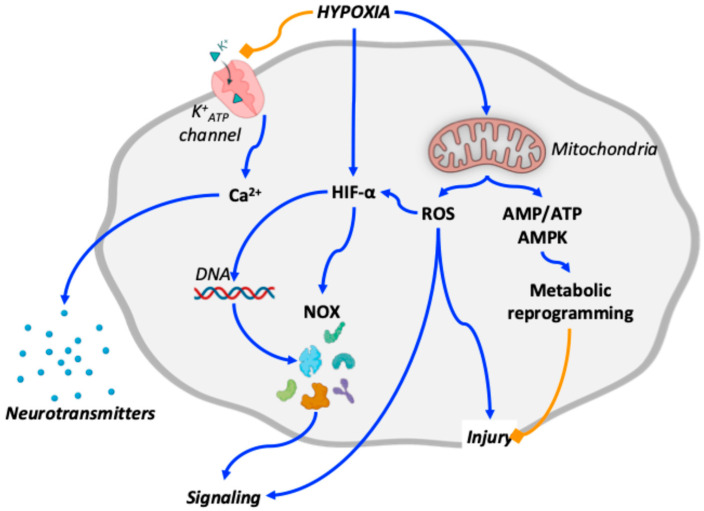
Schematic overview of oxygen-sensing mechanisms relevant to the cardiopulmonary system in altitude-induced and Group 3 pulmonary hypertension. Blue arrows indicate stimulatory interactions, whereas orange blunt-ended lines denote inhibitory effects. Hypoxia inhibits K^+^_ATP_ channel activity while activating hypoxia-inducible factors (HIFs) and mitochondrial signaling. Inhibition of K^+^_ATP_ channels promotes intracellular Ca^++^ transients that trigger neurotransmitter release. HIFs regulate the expression of multiple signaling mediators, including NADPH oxidases (NOX). Mitochondrial overactivation leads to increased production of reactive oxygen species (ROS), contributing to cellular injury, further stabilization of HIFs, and integration of downstream signaling pathways. In parallel, mitochondrial overactivation increases the AMP/ATP ratio, resulting in AMP-activated protein kinase (AMPK) activation and metabolic reprogramming, which may partially counteract ROS-induced injury. For clarity, additional coordinated pathways are not depicted and are discussed in the main text.

In the cardiopulmonary system, the main O_2_ sensing mechanisms triggered by hypoxia are summarized in Figure 1.

#### 2.1.1. HIF

The primary molecular response to inadequate O_2_ availability is the activation of hypoxia-inducible factors (HIFs). These transcription factors bind to hypoxia response elements within the promoter regions of target genes [16]. In concert with other regulatory proteins [17], HIF orchestrates the responses to hypoxia through the up- or down-regulation of hundreds of proteins [18]. Although HIFs belong to a family of at least three isoforms—HIF-1, HIF-2, and HIF-3— HIF-2α, also known as endothelial PAS domain protein 1 (EPAS-1), constitutes the isoform that is principally expressed in lungs [19].

#### 2.1.2. Mitochondria

Mitochondria serve not only as cellular powerhouses but also as key sensors of hypoxia, initiating adaptive responses through complex signaling networks that frequently converge with HIF pathways. Hypoxia influences mitochondrial function through at least three distinct mechanisms:

- Increased release of reactive O_2_ species (ROS) at complexes I and III of the electron transport chain due to mitochondrial uncoupling [20]. Besides inducing the morphological changes discussed below [21], excess ROS reduces the mitochondrial Ca^++^ uptake [22]. The consequent intracellular Ca^++^ overload not only brings injury to cell structures, but also contributes to PH by stabilizing HIF [23,24], increasing muscularity of pulmonary arterioles, and inducing contraction of pulmonary vessels [25].
- Increased activity and expression of NADPH oxidases (NOXs), a family of enzymes that generate ROS under the transcriptional control of HIF-1α [26]. Among the NOX isoforms identified in humans [27], NOX4 is particularly relevant, as it is directly induced by hypoxia [28] and represents a major ROS source in mammalian tissues [29]. Exposure of mice to simulated high altitude (5000 m for 4 weeks) resulted in an almost twofold increase in NOX4 expression in the brain, confirming its hypoxia responsiveness [30]. Mechanistically, NOX catalyze the transfer of electrons from NADH to O_2_, generating superoxide anions as primary ROS. Importantly, in COPD patients [31] NOX4 expression was related with PH severity, and its pathogenic role was further supported in experimental models of hypoxia-induced PH [32]. Conversely, activation of the nuclear factor erythroid 2-related factor 2 (Nrf2) serves as an adaptive counter-regulatory mechanism. Nrf2, a transcriptional antagonist of NOX4, is activated in hypoxia [33] and has the role of enhancing antioxidant defense by upregulating the expression of cytoprotective genes [34].
- Decreased ATP production by dysfunctional mitochondria [35] that raises the cell AMP/ATP ratio, thereby activating AMPK [36]. AMPK can also be activated by oxidative stress without the intervention of AMP [37]. Irrespective of the upstream mechanism, activated AMPK regulate downstream signaling pathways that contribute to the cell adaptation to hypoxia. First, AMPK improves cell energy conservation by inhibiting the anabolic processes that consume ATP, such as protein synthesis [38], or by upregulating the catabolic pathways, such as fatty acid oxidation [39]. Second, AMPK interacts with HIF by regulating directly the expression of genes that favor hypoxia adaptation [36].

#### 2.1.3. Oxygen-Sensitive Ion Channels

By inhibiting K^+^_ATP_ channels, O_2_-sensitive proteins embedded in cell membranes that play a crucial role in several cellular and physiological responses [40], hypoxia contributes to cell depolarization, which improves the Ca^2+^ influx and consequently neurotransmitter release or vasoconstriction, leading to HPV. Although primarily involved in the carotid body response to hypoxia, K^+^_ATP_ channels have been found in various cells and tissues, including PASMC [41].

### 2.2. Responses to Chronic Hypoxia

Although both acute and chronic hypoxia derive from insufficient O_2_ reaching the tissues, they involve distinct etiologies and pathogenic mechanisms. During acute hypoxia, HIF overexpression is generally believed to be protective, as it induces the overexpression of genes that promote cellular adaptation to low O_2_ availability—such as VEGF for angiogenesis, erythropoietin (EPO) for red blood cell production, and glycolytic enzymes for anaerobic metabolism. HIF-1α also contributes to maintaining alveolar epithelial barrier integrity, modulating inflammation, and facilitating vascular remodeling to enhance O_2_ delivery. By contrast, chronic hypoxia is often linked to the development of PH, which will be the focus of the following discussion.

#### 2.2.1. Lungs

Although the lungs are the first organ in the body to be exposed to hypoxia, our understanding of pulmonary responses to chronic hypoxia is still incomplete and, at times, contradictory. Sustained alveolar hypoxia is clearly associated with pulmonary vasoconstriction and HPV onset [42]. The nature of the hypoxic stimulus (e.g., acute or chronic), the specific cell type involved (e.g., PAEC, PASMC, or alveolar epithelial cells), and the predominant HIF isoform expressed (e.g., HIF-1α or HIF-2α) critically influence whether the response to hypoxia is adaptive or pathological.

PASMC mitochondria act as primary hypoxia sensors and HPV effectors [43], but some of the adverse responses depend on HIFs overexpression, which targets pulmonary remodeling through modulation of rho kinase [44] and VEGF [45]. Elevation of HIF-1α in human PASMC and of HIF-2α in PAEC indicates that HIFs play relevant pathogenic roles in severe PH [46]. Furthermore, HIF-1α inhibits the angiotensin-converting enzyme-2 expression, which contributes to HPV by stimulating PASMC proliferation and migration [47]. Even pulmonary fibrosis is triggered by HIF-1α through endoplasmic reticulum stress and pro-apoptotic transcription factor C/EBP homologous protein-mediated apoptosis in alveolar epithelial cells [48]. Remarkably, HIF-1α is also a key mediator in post-injury inflammatory processes [49] through inflammatory markers that activate either acutely through the NOD-like receptor 3 inflammasome [50], or chronically through the epidermal growth factor receptor/phosphoinositide 3-kinase (PI3K)/protein kinase b (Akt) pathway [51]. Finally, HIF-1α downstream proteins upregulate the VEGF pathway [52]. Taken together, such evidence indicates that pharmacological inhibition of HIFs, especially HIF-2α, might represent a promising therapeutic strategy for the treatment of vascular remodeling during PH [53]. Other studies, however, highlighted positive effects of HIFs in hypoxic lungs, but mostly in acute situations. For example, HIF-1α signaling promotes the repair of the alveolar epithelium after acute lung injury [54]. Likewise, therapeutic activation of HIF-1α-dependent vascular repair has been proposed as an effective therapy to treat inflammatory vascular diseases [55]. PAEC HIF-2α knockout prevents hypoxia-induced PH in mice, further suggesting that the inhibition of HIF-2α (but not of HIF-1α) can provide a therapeutic approach to treat hypoxia-induced PH [56]. Although hypoxia exacerbates inflammatory acute lung injury via the Toll-Like Receptor 4 signaling pathway, increasing HIF-1α enhances anti-inflammatory protection in rats [57]. Analyses of recent narrative reviews across pulmonary diseases consistently highlight the dual role of HIFs as both protective and pathogenic mediators [58,59,60,61]. On the one hand, HIF signaling promotes tissue repair, including vascularization, preservation of barrier integrity, and metabolic adaptation to hypoxia. On the other hand, sustained HIF activation enhances inflammatory responses by promoting pro-inflammatory immune cell phenotypes, such as M1 macrophages, and delaying neutrophil apoptosis, thereby exacerbating tissue injury. This functional duality complicates therapeutic targeting of HIF pathways and underscores the need for stage- and context-specific intervention strategies.

#### 2.2.2. Heart

One of the highest O_2_-consuming tissues, the myocardium, is highly sensitive to reduced O_2_ availability. Studies comparing chronic and intermittent hypoxia have provided insight into the underlying molecular mechanisms. Chronic hypoxia impairs cardiac performance and inhibits the development of endogenous protective responses to ischemic injury. By contrast, intermittent hypoxia activates cardioprotective pathways. Consequently, hypoxic conditioning emerged as a promising, though still underexplored, strategy for enhancing cardioprotection, particularly in populations residing at moderate altitudes. However, when hypoxia arises from pulmonary diseases such as PH, the resulting pressure overload leads to RV hypertrophy and eventually RV failure. In exploring the role of HIF signaling in these processes, it is notable that, at the same severity and duration of hypoxia, myocardial HIF-1α expression is less pronounced compared to that in brain, kidney cortex, skeletal muscle, and other organs [62]. Nonetheless, HIF-1α is rapidly induced within an hour of hypoxia in vivo [63]. Emerging evidence also indicates that HIF upregulation during early hypoxia is partially driven by inflammation-induced transcriptional changes, potentially mediated by O_2_-sensing prolyl hydroxylases (PHDs) that regulate HIF stability via proteasomal degradation [64]. Additionally, suppression of myocardial angiogenesis under hypoxia—through downregulation of HIF-1α and VEGF—has been shown to mitigate RV hypertrophy [65].

RV exhibits responses to hypoxia that differ from LV. In a PH-linked context, hypoxia affects the RV both directly by limiting myocardial oxygen availability, and indirectly by increasing pulmonary vascular resistance and afterload. Early RV adaptation is characterized by metabolic reprogramming (see Section 3.8) due to HIF activation [66]. In humans, RV metabolic reprogramming promotes not only metabolic flexibility, but also angiogenic signaling with preservation of capillary density and hence RV hypertrophy [67]. However, when sustained, hypoxia and pressure overload shift such adaptive mechanisms into maladaptive because persistent glycolytic reprogramming suppresses mitochondrial oxidative metabolism with increased redox imbalance (see Section 3.1). In parallel, chronic hypoxia promotes pulmonary arterial fibroblast (PAAF) activation and extracellular matrix deposition, thereby increasing RV stiffness (see Section 3.10). In addition, hypoxia-induced Ca^++^ overload, mainly driven by dysfunctional K^+^_ATP_ channel (see Section 2.1.3), further impairs contractile performance. Since exercise contrasts the deleterious effects of uncontrolled angiogenesis [67], it is expected that high-altitude adaptation exerts similar features. Thus, RV responses to hypoxia evolve from initially compensatory to maladaptive, highlighting the importance of RV-specific metabolic, angiogenic, and mitochondrial pathways as therapeutic targets in PH.

## 3. The Hypoxia–PH Axis

**Figure 2:**
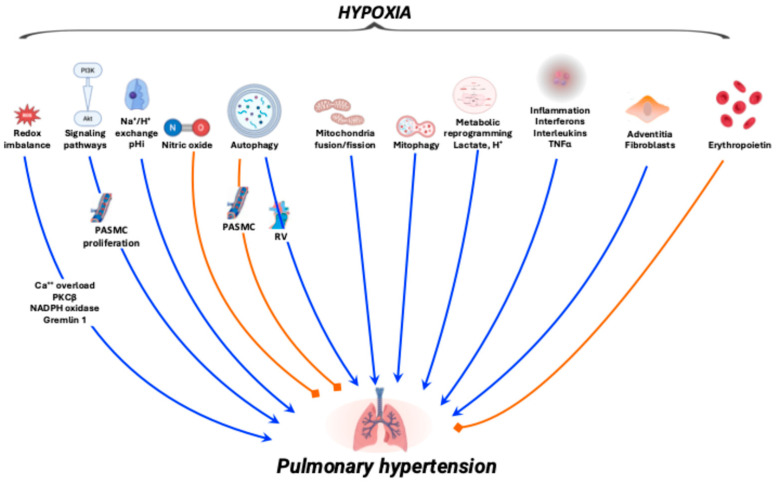
Schematic overview of the hypoxia-pulmonary hypertension axis. Blue arrows indicate the mechanisms leading to pulmonary hypertension, whereas orange blunt-ended lines denote inhibitory effects. Details on the various mechanisms are discussed in the main text.

Once established that O_2_ sensors in pulmonary and myocardial tissues activate downstream signaling pathways in response to hypoxia, the next step is to elucidate which of the downstream mechanisms become preponderant in raising protective or pathological outcomes with respect to PH (Figure 2).

### 3.1. Redox Imbalance

Chronic hypoxia-induced redox imbalance, whether arising from high-altitude exposure or pulmonary disease, is well recognized as a key contributor to HPV and PH, primarily through intracellular Ca^2+^ overload [68,69]. Ca^2+^ overload and redox imbalance are linked to PH through interconnected pathways involving transient receptor potential cation channel subfamily V member 4 (TRPV4), mitochondrial ROS, protein kinase C beta type (PKCβ), and NOX-dependent signaling. These cascades converge on structural remodeling of the pulmonary vasculature through the following mechanisms:

- Ca^++^ influx and contractility in PASMC, by TRPV4 channel activation or contractile potentiation. Hypoxia enhances Ca^++^ entry through TRPV4, as shown in Sugen-hypoxia (SuHx) models [70]. The resulting intracellular Ca^++^ rise augments PASMC contractility, thereby amplifying HPV [71,72,73]. ROS-dependent Ca^++^ influx was indeed identified as a target in conditioning medicine, offering a potential avenue for interventions [74].
- Intracellular Ca^++^ release and cytoskeletal signaling. This action can be mediated by the activation of either the ryanodine receptor-2 or Rho kinase pathways. While the sarcoplasmic reticulum contributes to HPV through Ca^++^ release mediated by ryanodine receptor-2 channels [75], actin polymerization and cytoskeletal reorganization, driven by Rho kinase activation, further sustain PASMC contraction and vascular remodeling [76].
- ROS-dependent mitochondrial and cytosolic signaling driven by PKCβ: In PASMC from hypoxic neonatal rats, PKCβ enhances mitochondrial ROS production, reinforcing vasoconstriction and remodeling [77]. NOX also contributes because in pulmonary artery fibroblasts, the antifibrotic agent pirfenidone mitigates hypoxia-induced PH by inhibiting the NOX/ROS/p38 mitogen-activated protein kinases (MAPK) signaling cascade [78].
- Gremlin-1-mediated redox signaling and vascular remodeling. Gremlin-1, a known inhibitor of the transforming growth factors beta (TGFβ) pathway, contributes to PH pathogenesis and represents a potential therapeutic target in congenital heart disease-associated PH [79]. Endothelial NOX1 activity promotes Gremlin-1-dependent proliferation of PASMC, accompanied by increased ROS generation [80]. In human PASMC, NOX1 oxidase further stimulates Gremlin-1-driven cell proliferation and migration under hypoxic conditions [81].

Should ROS be a prominent cause of PH, then treatments with antioxidants are expected to be efficient. A variety of antioxidants are indeed beneficial in several animal models [82], for example, mitochondrial thioredoxin 2 [83]. Furthermore, pro-oxidant co-morbidities such as diabetes are well known to exacerbate mitochondrial ROS in PAEC [84]. Likewise, the senescence of pulmonary fibroblasts synergizes with PH pathogenesis via a ROS-linked mechanism [85]. Remarkably, antenatal administration of the antioxidant melatonin efficiently enhances the redox balance in the postnatal lung in a PH newborn sheep model [86]. Additionally, the superoxide dismutase mimetic and peroxynitrite scavenger MnTBAP, a synthetic metalloporphyrin, reverses pulmonary vascular remodeling and improves cardiac function in a SuHx model [87]. Finally, the effects led by excess glucocorticoid and dexamethasone, which promote cardiac dysfunction and PH, are blunted in mice deficient in p22phox-dependent NOX, perhaps through a mechanism involving HIF-1α [88]. By contrast, 17β-estradiol and 2-methoxyestradiol ameliorate hypoxic PH by increasing manganese superoxide dismutase activity, a strong endogenous antioxidant [89]. Another steroid-derived molecule, andrographolide, a potent anti-inflammatory agent with antioxidant activity, attenuates PH through modulation of NOX/Nrf2-mediated oxidative stress and NF-κB-mediated inflammation [90]. However, despite such strong preclinical support, the clinical translation of antioxidants to treat PH is still limited. Few clinical trials have evaluated antioxidants either as monotherapy or adjuncts in PH patients, with the result that the favorable outcomes observed in preclinical studies have not been consistently reproduced in clinical practice. Although studies with N-acetyl cysteine—an FDA-approved antioxidant from L-cysteine used to treat various lung diseases—and other simple antioxidants have shown some improvement in endothelial function and biomarkers in PH and PAH patients, such an outcome is not being considered a primary clinical endpoint. Indeed, the usual problem in the treatment of PH is that preclinical studies in animals often do not represent the real human disease. Therefore, antioxidants may only address a minor part of the disease process and in human PH patients it remains difficult to show a real change. Thus, despite remarkable support in animal models, most current therapies do not directly target the redox imbalance.

### 3.2. The PI3K-Akt Pathway

The PI3K-Akt signaling pathway promotes survival in response to various extracellular factors that enhance PI3K phosphorylation. The PI3K isoform p110α is the main isoform that promotes cell growth by phosphorylating Akt at Thr^308^ and Ser^473^ residues. Phosphorylated Akt inhibits apoptosis and stimulates protein synthesis through the activation of cell surface receptors and the formation of the second messenger phosphatidylinositol (3,4,5)-trisphosphate [91]. At present, Akt is known to phosphorylate as many as 100 different substrates, leading to a wide range of effects in most mammal cells [91]. In contexts related to PH, the PI3K-Akt axis includes the mammalian target of rapamycin (mTOR) to critically regulate cell metabolism and RV function during hypoxia. This reveals relevant therapeutic opportunities. For example, luteolin, a plant-derived flavonoid, restores PI3K, Akt, and mTOR phosphorylation improving RV structure and function in PH rats [92]. Knockout of some lipid-metabolizing enzymes, such as arachidonic acid 15-lipoxygenase and its isoforms, which are upregulated in several experimental PH models, attenuates pulmonary vascular remodeling with improvement of RV structure and function through modulation of the PI3K-Akt-mTOR signaling [93]. Finally, the Inhibition of C1q/tumor necrosis factor-related protein 1 (CTRP1), an adipokine secreted by adipose tissue that regulates glycolysis and is upregulated in PH mice, markedly improves mPAP and RV function through PI3K-Akt-mTOR inhibition [94].

The upregulation of the PI3K-Akt pathway has been linked to PH in monocrotaline-induced PAH rats, where this pathway increases with PAH progression and correlates with pro-inflammatory proteins [95]. Efforts are being made to develop inhibitors and antagonists of this pathway. The disruption of p110α in PASMC indeed blunts in vitro responses and prevents/reverses pulmonary vascular remodeling, PH, and RV hypertrophy in chronic hypoxia and SuHx models [96]. Phosphoinositide-dependent protein kinase-1, a hypoxia-responsive protein in stress responses [97,98], serves a key role in Akt activation [99]. Partial knockout of phosphoinositide-dependent protein kinase-1 reduces hypoxia-induced Akt activation and PH [100]. The PI3K-Akt pathway might be one of the potential biomarkers for the severity of PH [101]. Akt revealed protective features in chronic situations, possibly overriding the protection elicited by Nrf2 [30].

### 3.3. Na^+^/H^+^ Exchange

The intracellular pH (pHi) is under the control of several systems that, besides the Na^+^/HCO_3_^−^ cotransport and the Cl^−^/HCO_3_^−^ exchanger, involve the Na^+^/H^+^ exchanger (NHE). NHE is a ubiquitous membrane passive antiporter protein driven by the Na^+^ gradient formed by Na^+^/K^+^ ATPase [102]. At least nine isoforms were found in human and rat tissues, with human cardiomyocytes mainly expressing NHE-1 [103]. Essentially inactive at neutral pHi, NHE-1 is activated either by intracellular acidosis, a typical outcome of myocardial damage, or by myocardial stretch, as in hemodynamic overload [104,105] as well as in RV or left ventricle (LV) hypertrophy [106]. In the LV of spontaneously hypertensive rats, NHE-1 is activated by phosphorylation of the Ser^703^ residue, one of the targets of Akt signaling [107]. Both gene and protein NHE-1 expression increase in chronically hypoxic mouse PASMCs [108]. Although the role of HIF-1 in this process appears essential [109], NHE-1 upregulation in hypoxic cardiomyocytes may also be due to intracellular acidosis secondary to the onset of anaerobic glycolysis [110]. The vasoconstrictor endothelin-1, often upregulated in several forms of PH, may contribute to increased NHE activity in rat PASMC favoring, PASMC migration, and proliferation through a pathway involving rho kinase [111].

Several NHE inhibitors are under scrutiny in the search of cardio-protective drugs in models of ischemia–reperfusion and transplantation [112,113,114,115]. Among these, cariporide and eniporide reached Phase 2/3 in clinical trials [116,117,118]. Either inhibitor, however, did not elicit significant positive effects, despite their efficacy in animal models. Either cariporide or empagliflozin, a Na^+^/glucose co-transporter 2 inhibitor, downregulates NHE-1 expression in human atrial and mice ventricle myocytes [106]. Although the effects of various NHE inhibitors were studied in cardiomyocytes or pulmonary vasculature, to date the effects of NHE-1 expression on hypoxia-induced RV hypertrophy are rather obscure. While a study focused in cariporide, which abrogated the NHE-1 overexpression in the RV of rats with monocrotaline-induced PH [119], another study highlighted the role of rimeporide, which prevents functional, morphological, and biochemical deleterious effects of PAH in both the RV and lungs [120], thereby pointing at this drug as an efficient therapy to treat PAH.

### 3.4. Nitric Oxide

Nitric oxide (NO) brings vasodilation in hypoxic systemic and pulmonary vessels [121]. The NO system is involved in clinical and AIPH. PAH patients have low NO levels in the broncho-alveolar lavage fluid [122]. Lower NO in exhaled air correlates with structural damage to pulmonary arteries and to reduction in the PAEC. NO synthesis is reduced in persistent PH in the newborn along with low plasma levels of L-Arginine (L-Arg), which suggests that inadequate NO production is a major contributor to pulmonary pathogenesis [123]. This outcome may derive from either depressed activation [124] or protein expression downregulation of the endothelial isoform of NO synthase (eNOS) in pulmonary arteries, as shown in COPD patients [125] and smokers [126]. Mice knocked out for caveolin-1, a membrane protein involved in endocytosis, exhibit suppression of eNOS activity with increased RV systolic pressure and pulmonary vascular remodeling [127].

NO is a druggable target in clinical PH. As Group 3 PH patients display low plasma L-Arg levels [128,129], its supplementation attenuates PH in hypoxia-challenged rats [130] and reduces pulmonary vascular resistance [131]. In pigs, L-Arg was successfully replaced by the combination of L-citrulline with tetrahydrobiopterin [132]. Besides providing the substrate for eNOS, in PAEC L-Arg also prevents eNOS uncoupling, which generates reactive nitrogen species (RNS) [133]. NO donors can hyperpolarize PASMC with subsequent pulmonary artery vasodilation by enhancing voltage-gated K^+^_ATP_ channels currents [134].

NO is tightly linked to both acute and chronic altitude PH. In acute conditions, HPV is correlated to decreased pulmonary NO [135,136,137], NO inhaling can be used therapeutically to improve arterial O_2_ saturation in pulmonary edema patients [138]. In subacute conditions, after an initial (hours) fall of NO levels in lungs and plasma, NO tends to return toward and beyond baseline levels in the following days of hypoxia [139]. For longer (months) hypoxia durations, higher plasma L-Arg, suggestive of high plasma NO levels, was observed in dwellers at 13% O_2_ for 10 months [140], according to the beneficial effects of supplementing L-Arg [141] or sildenafil [142] at altitude. Finally, for very long (generations) hypoxia, Tibetans display higher NO levels in the lung and plasma than any other highland population [139]. If the Tibetan population is assumed to be altitude-adapted, then the observation that their blood has high plasma NO-storing capacity may be functional to highlight an important role of NO in altitude adaptation, or sporadic CMS symptoms [143,144]. Reduced erythropoiesis in Tibetans, with lower diastolic blood pressure, is another feature that results from a marked vasodilatory response to hypoxia in adapted individuals [145]. Remarkably, 14–20% of the Kyrghyz commuters show signs of AIPH [10], probably for the occurrence of genetic traits [146]. Improved NO bioavailability appears thus to favor altitude adaptation [147].

Mechanistically, the vasodilatory effect of NO is brought about by its binding to soluble guanylate cyclase, which favors the buildup of cyclic guanosine monophosphate (cGMP), which upregulates myosin phosphatase and smooth muscle relaxation. This process is terminated by a phosphodiesterase (PDE) that hydrolyzes a chemical bond in cGMP, converting it to inactive 5′-GMP. Of the eleven known PDE families [148,149], the most relevant here is PDE5, whose best-known inhibitor is sildenafil, with its related compounds tadalafil and vardenafil. The relative abundance of PDE5 in PASMC provides the molecular basis for the use of PDE5 inhibitors in the treatment of PAH [150], especially in children [151]. Sildenafil can alleviate symptoms not only in PH, but also in several other cardiopulmonary diseases in a manner devoid of remarkable adverse effects [152]. In addition, it can correct hypoxia-induced RV hypertrophy [153]. Besides favoring vasodilatation, the sildenafil-induced increase in cGMP also upregulates eNOS Ser^1177^ phosphorylation, which in turn activates Akt with alleviation of intracellular Ca^2^ overload via mitochondrial K^+^_ATP_ channels opening [154] and reduction in cardiomyocytes apoptosis, thereby raising cardioprotection in chronically hypoxic hearts [155]. Remarkably, the mechanisms elicited by sildenafil persist over time for at least 4 weeks [156]. In COPD patients, despite poor evidence for the long-term clinical benefits of PDE5 inhibitors [157], sildenafil could ameliorate the quality of life by reducing pulmonary vascular resistance and improving body mass index, airflow obstruction, dyspnea, and exercise capacity [158]. PDE5 inhibitors may affect directly pulmonary remodeling. In a model of hypoxia PH, sildenafil increased the abundance of small (<50 μm diameter) pulmonary vessels, leaving large vessels nearly unaffected, which translates into marked effect of sildenafil in downregulation the hypoxia-induced formation of new vessels [159]. The mechanisms underlying this phenomenon may involve the suppression of the hypoxia-induced increase in c-kit^+^ cells, which colocalize with VEGF receptor 2 and CD68, and moderate hypoxia-induced PASMC proliferation and inflammation [160]. Another key mechanism recruited by sildenafil concerns the basal lamina, a layer of extracellular matrix secreted by lung epithelial cells that serves both as an attachment point for cells and as a permeability barrier. Its thickness represents a compromise between the need to provide greater mechanical resistance against pressure, a highlight in PH, and to facilitate gas diffusion across the alveolar-capillary barrier [161]. This subtle compromise is overruled in instances such as diabetes mellitus, asthma, and COPD that thicken the basal lamina with slower gas passage across the alveolar-arterial barrier [162,163]. By contrast, the greater fragility of the barrier in pulmonary edema has been attributed, at least in part, to failed basal lamina thickening [164]. Sildenafil could restore the thickening of the basal lamina observed in a hypoxia-PH rat model, highlighting a novel effect of PDE5 inhibition in hypoxic PH [159]. Few studies have compared the effects of the various NO therapeutics. However, in a model of hypoxia PH, treatments based on sildenafil, L-Arg, and molsidomine were virtually indistinguishable as all the mechanisms have an anisotropic effect in reducing RV pressure [156].

### 3.5. Autophagy

Autophagy, a physiological process of intracellular protein degradation, is appearing as a major player in PH development. The autophagy process maintains cellular homeostasis by recycling redundant, damaged proteins and organelles through lysosome-dependent degradation [165]. Microautophagy, chaperone-mediated autophagy, and macroautophagy, the main forms of autophagy [166], involve multiple proteins systems, but microtubule-associated protein light chain 3 (LC3-II), Beclin-1, and sequestosome 1 (p62) are the main keys to characterize the autophagic flux: increased Beclin-1 indicates initiation, reduced p62 indicates autolysosome degradation, and increased LC3-II indicates autophagosome maturation [167].

In the lungs, the autophagy process concerns mainly PAEC and PASMC. LC3-II is supposed to play a highly specific protective role during PH development, but this view still lacks definitive confirmation. On one hand, both PH patients and mice exposed to chronic hypoxia for 3 weeks showed increased lung LC3-II [168]. Furthermore, LC3-II (-/-) mice displayed exaggerated PH in chronic hypoxia [168]. On the other hand, in a monocrotaline rat model of PH, the disease progression is associated with increased LC3-II and decreased p62 [169], with LC3-II suppressing PASMC proliferation [170]. LC3-II expression is also increased in the lungs of COPD patients [171] and of cigarette smoke-sensitized mice [172]. Hypoxia-activated autophagy induces PAEC proliferation and apoptosis in precapillary pulmonary arterioles [173]. By promoting PASMC proliferation and migration, the latter mechanism contributes to PH development through induction of distal vessel muscularization. Autophagy is also selectively involved in RV failure. The level of LC3-II is elevated in the RV, but not in the LV of SuHx rats [174]. By controlling collagen degradation, RV autophagy has a relevant role in myocardial fibrosis [175], which is linked to RV hypertrophy [176]. The paths leading to autophagy are probably intersected with those related to NHE in both in vitro model of cardiac hypertrophy [177] and SuHx rats [120]. Finally, the small GTPase RAB7A, which has a key function during the fusion of lysosome to autophagosome [178], is reduced in PAEC but not in PASMC from PH patients, and endothelial-specific reduction in RAB7A expression can cause PH in mice [179]. Treatment with RAB7 GTPase activator ML-098 reduces severe PH in SuHx rats [179].

There is evidence that altitude hypoxia induces autophagy. For example, high-altitude Tibetans with congenital heart disease are able to resist ischemia–reperfusion injury during cardiac surgery better than their respective low-altitude counterparts, possibly through elevated basal autophagy induced by chronic hypoxia, as measured through myocardial LC3-II, which was higher in the high- vs. low-altitude group [180]. Exposure to high-altitude hypoxia for long periods results in lung injury aggregation, formation of autophagosomes with a double-membrane structure and increased levels of Beclin-1 and LC3-II in alveolar tissues [181]. Finally, treatment with Eleutherosides, a group of chemical compounds found in *Eleuthoerococcus senticosus*, also known as Siberian ginseng, has anti-inflammatory and antioxidant effects through the restoration of impaired autophagic flux by activating the AMPK/mTor signaling pathway [182]. In the high-altitude yaks, the expression of HIF-2α, Beclin-1 and LC3-II in the cerebral cortexes, and hippocampi was higher than those in low-altitude yaks [183], indicating that autophagy may enhance brain tissue adaptation to hypoxia condition.

### 3.6. Mitochondrial Fission and Fusion

The observation that pulmonary diseases alter two of the three major lipid classes of the inner mitochondrial membrane, phosphatidylcholine, phosphatidylethanolamine, and cardiolipin [184], strongly supports a pivotal role for mitochondria [185]. Mitochondria are central to both acute and chronic O_2_ sensing and hypoxic adaptation [20]. A detrimental feedback loop is indeed established when damaged mitochondria increase ROS production, which in turn promotes lipid peroxidation and further mitochondrial damage. The detection of cell-free mitochondrial DNA in plasma has been associated with disease severity and progression in COPD [186,187] and acute respiratory distress syndrome (ARDS) [188] patients, addressing cell-free mitochondrial DNA levels in plasma as a biomarker of anti-inflammatory treatment efficacy in ARDS [189].

Mitochondrial fission and fusion, dynamic processes that regulate mitochondrial morphology, are primary components of the body’s responses to hypoxia. Fission is the division of a single mitochondrion into two or more, while fusion is the merging of two or more mitochondria into one. These stress-sensitive processes are crucial for maintaining mitochondrial health and influence various cellular functions. Hypoxic cells experience increased mitochondrial fission and decreased fusion, which lead to mitochondrial dysfunction [190,191]. The underlying molecular mechanisms stem from HIF-1α stabilization that activates dynamin-related proteins [192]. Mitochondrial fragmentation may modulate ROS-dependent Ca^2+^ overload [193]. Hypoxia activates a set of redox-independent counter-regulatory mechanisms aimed at preserving mitochondrial integrity, limiting ROS-mediated injury, and maintaining bioenergetic homeostasis. Several coordinated mechanisms are involved, some of which may result in profitable therapeutic targets in PH:

- The overexpression of Sirtuin1, a NAD-dependent deacetylase, regulates mitochondrial function [194] and exerts protective effects in experimental models of PH by mitigating oxidative injury [195,196]. Sirtuin1 also restores the mitochondrial NAD^+^/NADH balance, regulates mitochondrial homeostasis, and counteracts PASMCs migration and proliferation [197].
- HIF-driven metabolic reprogramming with upregulation of glucose utilization and of glycolytic enzymes. As discussed in Section 3.8, metabolic reprogramming reduces oxidative phosphorylation, thereby relieving the pressure on the electron transport chain and reducing ROS leakage. As a matter of fact, persistent activation of HIF-1α in PASMCs and of HIF-2α in PAECs, even in normoxia [198], leads to a Warburg-like phenotype that promotes the metabolic backbone of PH, i.e., PASMC hyperproliferation, apoptosis resistance, and vascular remodeling. However, the identification of this axis may open promising avenues in the treatment of PH based on HIF inhibitors [199].
- Increased hypoxia-induced mitophagy as a key to improving mitochondrial quality control by eliminating dysfunctional mitochondria before they trigger apoptosis, as discussed in Section 3.7. While in physiological hypoxia, HIF-1α overexpression promotes the expression of BNIP3 and NIX—mitochondrial proteins that act as key receptors for mitophagy [200] —the expression of those proteins is blunted in PH [201]. Consequently, damaged hyperpolarized mitochondria accumulate instead of being removed, leading to altered redox signaling and resistance to mitophagy.
- Mitochondrial biogenesis tuning to replace damaged mitochondria, mainly modulated by peroxisome proliferator-activated receptor gamma co-activator-1α (PGC-1α). Originally identified as a key regulator of energy metabolism [202], suppressed in acute hypoxia [203] but reactivated during chronic hypoxia or recovery [204], PGC-1α-mediated angiogenesis prevents PH in mice [205]. Attempts to restore PGC-1α expression may offer new therapeutic targets, at least in persistent PH of the newborn [206]. Mitochondrial dynamics may be controlled by alternative mechanisms discussed in Section 3.7.

### 3.7. Mitophagy

Mitophagy, a type of autophagy selective for mitochondria, is a crucial cellular process activated under hypoxic conditions to maintain mitochondrial quality and homeostasis. This process controls mitochondria abundance rather than shape and dimension. Only way for removing selectively entire mitochondria, mitophagy is thus responsible for the correct mitochondria turnover [207]. Despite successful identification of several mitophagy-associated proteins, the exact molecular mechanisms are still poorly understood. Mitophagy is initiated by a change in mitochondrial membrane potential that promotes the accumulation of PTEN-induced kinase 1 (Pink1) on the outer mitochondrial membrane, which leads to the recruitment of cytoplasmic Parkin and ubiquitination of damaged mitochondria [208]. The ubiquitination of mitochondrial outer membrane proteins leads to the activation of mitophagy through several autophagy receptors, some of which are triggered by hypoxia [209]. The uncoupling protein 2 (UCP2) localized in the inner membrane of mitochondria is involved in mitophagy because its loss increases the levels of mitophagy-associated proteins Pink1 and Parkin inducing spontaneous PH [210]. The failing RV exhibits signals that address the onset of mitophagy: reduction in mitochondria number, size, and shape abnormalities accompanied by metabolic changes that include uncoupled glycolysis and impaired fatty acid utilization. PH models also display abnormalities in terms of mitochondria swelling [211,212] and dysfunction [201,213,214]. As an inhibitor of mitophagy, cyclosporine indeed reduces PASMC proliferation in hypoxic cells [215]. Likewise, mice knocked down for UCP2, an anion transporter in the inner mitochondrial membrane that regulates mitophagy, exhibit worse hypoxic PH related to mitochondrial hyperpolarization [216]. Loss of UCP2 in endothelial cells increases mitophagy, decreases mitochondrial biogenesis, and increases apoptosis [210].

Mitophagy may be mediated by several receptors that include FUN 14 domain-containing 1 (FUNDC1), a receptor localized to the outer mitochondrial membrane that interacts with LC3 to recruit membranes for mitophagy [217]. Mitophagy upregulation causes PASMC proliferation through the ROS-HIF-1α pathway, which leads to pulmonary vascular remodeling. Inhibiting FUNDC1-mediated mitophagy genetically or pharmacologically could indeed be a therapeutic target for hypoxic PH as it ameliorates PASMC proliferation and pulmonary vascular remodeling [209]. However, to the best of our knowledge, no study has investigated the role of this receptor in the RV during hypoxic PH. In the RV of monocrotaline rats, the accumulation of the mitophagy markers Parkin and the phosphorylated derivative of dynamin-related protein 1 (DRP1), and the decrease in MFN2, indicate activation of mitophagy [218], which was restored by treatment with the anti-hypertrophic agent 1,8-cineole (a monoterpene oxide), which also restored the gap junction protein connexin-43 distribution at intercalated disk and SERCA2a protein levels, along with RV function improvement [218]. The expression of histone methyltransferase was significantly increased in the PASMC of chronically hypoxic rats/mice and in PH patients, while the inhibition or knockdown of SET and MYND domain-containing 2 (SMYD2), a methyltransferase that modifies other proteins by adding a methyl group, alleviated PH remodeling [219]. Mechanistically, SMYD2 aggravates PASMC proliferation and PH development by monomethylating the peroxisome proliferator-activated receptor gamma (PPARy)—a regulator of the genes involved in glucose homeostasis, fatty acid metabolism, and adipocyte differentiation—thereby enhancing PPARy-regulated mitophagy and disrupting mitochondrial energy metabolism [219].

### 3.8. Metabolic Reprogramming

Primarily caused by mitophagy dysfunction, the decreased mitochondria function results in complex metabolic reprogramming, with glycolysis surging as pivotal to supply anaerobic ATP. Originally identified in cancer cells as the Warburg effect, the glycolytic switch describes an increasing reliance of cell bioenergetics on glycolysis, which spares the mitochondrial function [220]. The discovery that the Warburg effect is activated by HIF-1α [221] supports the notion that the glycolytic switch is a consequence of the cell hypoxia state, a common feature both in carcinogenesis and in various cell lineages of PH lungs: PASMC, PAEC, and fibroblasts [213]. Studies in PH patients [222] and a meta-analysis of PH experimental models [223] highlighted changes in substrate utilization by the RV that correlate with disease severity and are compatible with the recruitment of the glycolytic switch as a tool to spare fatty acid oxidation. Survival of hypoxic cancer cells is associated with high fatty acid synthesis [224] and oxidation [225]. Likewise, in rat PH models and in human hypoxic PASMC, inhibition of fatty acid synthase by siRNA increases apoptosis, thereby limiting RV pressure, hypertrophy, and pulmonary vascular remodeling [226]. Targeting malonyl CoA decarboxylase to inhibit fatty acid oxidation is also protective in vascular smooth muscle cells [227]. The occurrence of the Warburg effect in the RV of SuHx rats was confirmed by nuclear imaging techniques that highlighted altered glucose utilization [228,229]. The RV of SuHx rats also displayed a marked decrease in malic and fumaric acid, as well as of long-chain acylcarnitines, which are essential to transport acyl groups to the mitochondria [230].

The glycolysis releases lactate and H^+^. Although high H^+^ reflects in reversible effects in isolated perfused hearts, high lactate irreversibly damages O_2_ utilization paths by at least two mechanisms: (a) impairment of phosphorylation coupling efficiency due to the dissipation of the H^+^ gradient across the inner mitochondrial membrane via upregulation of the lactate-H^+^ cotransport, and (b) lactate-induced increased amplitude of the Ca^++^ transients that leads to increased intracellular Ca^++^ load with higher costs required for pumping out Ca^++^ [231]. As lactate exportation out of the cell is limited, the intracellular buildup consequent to the Warburg effect acidifies the cell milieu, with side effects that may be noxious. A consequence of the glycolytic switch, the hyperpolarization of the inner mitochondrial membrane halts the release of pro-apoptotic factors [215,232], a hallmark of PH. Furthermore, the release and the accumulation of cytosolic survivin, a caspase inhibitor, promotes resistance to apoptosis [233], which downregulates vascular remodeling [234], a relevant process in hypoxic PH [159]. An underlying mechanism shared in both carcinogenesis [235] and in PH [228] includes the downregulation of the apoptotic potential via promotion of the pentose phosphate pathway, which enhances the antioxidant reserve.

### 3.9. Inflammation

The immune system is crucial for maintaining tissue homeostasis. During the response to injury, early recruitment of immune cells is orchestrated by chemokines and inflammatory mediators released from the local microenvironment. These infiltrating cells secrete pro-inflammatory cytokines such as interferon-γ (IFN-γ), interleukin (IL)-1β, and IL-6 during the first stages of inflammation. In contrast, at later stages of tissue repair, immune cells predominantly release anti-inflammatory mediators including arginase-1, TGFβ, and IL-10, which stimulate cell proliferation, extracellular matrix synthesis, and angiogenesis, thereby promoting tissue regeneration.

Inflammatory activation within the lung and pulmonary vasculature is recognized as a hallmark of PH [236,237]. Infiltration of bone marrow-derived monocytes and macrophages represents a major component of the inflammatory response surrounding pulmonary arteries during vascular remodeling [160]. Elevated circulating levels of pro-inflammatory cytokines, including IL-1β, IL-6, IL-8, and tumor necrosis factor-α (TNFα), have been consistently observed in both preclinical models and patients with PH [238,239], as well as in individuals with high-altitude pulmonary edema [240]. Notably, increased IL levels correlate positively with disease severity and cardiac biomarkers such as C-reactive protein and N-terminal prohormone of brain natriuretic peptide, suggesting its prognostic value in PH [241].

Pulmonary vascular remodeling in AIPH and Group 3 PH is sustained by a self-perpetuating crosstalk between stromal cells and multiple immune cell populations [242]. Disruption of this interaction may therefore represent a promising therapeutic strategy in PH contexts characterized by inflammation and fibrosis [243]. As discussed in Section 3.10, PAAF plays a central role in establishing a dynamic signaling network that promotes and stabilizes pulmonary vascular remodeling. Recruited immune cells further reinforce stromal activation and fibrotic responses. In particular, macrophages activate PAAF through the release of several growth factors, with a prominent role for the transforming growth factor-β (TGFβ), thereby enhancing extracellular matrix deposition and angiogenic responses [244]. Mast cells contribute proteases and vasoactive mediators that further remodel the extracellular matrix [244], while adaptive immune cells—especially Th17 cells [245]—promote PAAF proliferation and inflammatory signaling. Notably, both PAAF and immune cells undergo a metabolic shift toward glycolysis accompanied by increased NOX-dependent ROS production [246], amplifying stromal-immune crosstalk through shared metabolic and redox reprogramming.

Targeting inflammation offers promising therapeutic avenues for PH. Among chemokines, monocyte chemoattractant protein-1 (MCP-1/CCL2) exhibits the highest affinity for the C-C chemokine receptor 2 (CCR2). Under hypoxic conditions, activation of the CCL2/CCR2 signaling axis between resident and recruited lung interstitial macrophages contributes to PH pathogenesis by promoting thrombospondin-1 release and subsequent activation of TGFβ [239]. Therapeutic blockade of this pathway—either through CCL2-neutralizing antibodies or CCR2 disruption—attenuates monocyte recruitment, reduces vascular remodeling, and ameliorates hypoxia-induced PH in animal models [247]. Similarly, in humans ascending from low (225 m) to high altitude (3500 m), increased plasma thrombospondin-1 and TGFβ levels were normalized by anti-inflammatory treatment with dexamethasone [239]. Cell-based therapies employing mesenchymal stem cells (MSCs) have also emerged as promising interventions for both acute and chronic lung diseases due to their potent immunomodulatory and anti-inflammatory properties. These effects are largely mediated by the MSC secretome, which includes soluble cytokines and extracellular vesicles [248,249,250]. Exosomes, a subclass of extracellular vesicles with 30–150 nm in diameter, serve as key mediators of intercellular communication. MSC-derived exosomes have been shown to reverse experimental PH by shifting macrophage polarization from a pro-inflammatory to an anti-inflammatory phenotype and by decreasing IL-6 and TNFα levels [251,252]. Furthermore, engineered exosomes designed for targeted delivery to PAEC represent a promising strategy to enhance tissue-specific therapeutic efficacy [253,254].

### 3.10. Adventitia and Adventitial Fibroblasts

Not a mere passive structural layer, PAAFs have been recognized as a critical driver of vascular remodeling in PH [255]. Accumulating experimental and clinical evidence supports an “outside-in” paradigm whereby adventitial changes precede and promote medial and intimal remodeling, particularly in AIPH and Group 3 PH [256]. The adventitia is uniquely positioned to sense distinct environmental changes, from hypoxia to mechanical stress and inflammatory stimuli. The adventitia layer harbors a heterogeneous population of fibroblasts, progenitor cells, immune cells, and vasa vasorum, making it an active signaling compartment rather than a mere scaffold. In several experimental models of PH, adventitial thickening and PAAF activation occur before overt PASMC hypertrophy [255].

Quiescent PAAF are switched to an activated state at the onset of either hypoxia or inflammation in idiopathic pulmonary fibrosis [257] and PH [258]. HIF signaling, redox imbalance, mechanical stretch, growth factors such as TGFβ, PDGF, and endothelin-1 are key drivers of this activation [259], which implies PASMC proliferation, resistance to apoptosis, and metabolic reprogramming [260]. By secreting cytokines and chemokines that recruit and retain macrophages, mast cells, and lymphocytes within the adventitial space, activated PAAFs thus play a central role in organizing vascular inflammation [261]. As a result, a chronic, non-resolving inflammatory microenvironment that perpetuates PAAF activation and promotes their crosstalk with PASMCs and PAECs is finally established. As an additional hallmark of PAAF in PH, increased expression of NOX4 leads to sustained ROS production [262], which not only adds the toxic component, but also reinforces PAAF activation, stimulates PASMC proliferation, and contributes to PAECs dysfunction.

PAAF also mediate extracellular matrix remodeling [263,264]. Increased collagen and fibronectin deposition, elastin fragmentation, and enhanced lysyl oxidase-mediated crosslinking result in progressive vascular stiffening, a hallmark in both AIPH and Group 3 PH [265]. Arterial stiffness may act as a pathogenic signal itself, amplifying the pathways that further activate PAAF and PASMCs through a process known as mechanobiological feedback [266].

Adventitial mechanisms appear particularly prominent in both AIPH and Group 3 PH. While PAAF involvement in Group 3 PH is widely recognized, chronic exposure to hypoxia directly activates PAAF, thereby sustaining inflammation, fibrosis, and metabolic dysregulation [267]. A possible role for hypoxia-induced exosomes on PAAF metabolism in idiopathic pulmonary fibrosis was identified [268]. Thus, the role of adventitia and PAAFs as central orchestrators of pulmonary vascular remodeling in both AIPH and Group 3 PH is definitely ascertained.

### 3.11. Erythropoietin—Can It Be a Protective Factor?

Erythropoietin (EPO) is primarily recognized as an erythropoietic agent that stimulates the bone marrow to produce red cells [269] in response to hypoxia [270], thereby improving the O_2_ transport triad [271]. By targeting not only the erythroid but also neuronal cells, EPO is also neuroprotective [272,273]. Its therapeutic use in hypoxia is challenged by its synergy with hypoxia to excessively elevate hematocrit with potentially toxic effects [274]. However, by retaining the neuroprotective effect without increasing the hematocrit, non-erythropoietic EPO derivatives proved useful in reducing neuronal apoptosis in hypoxia-challenged rats [275]. The human EPO receptors are expressed not only in the erythroid and central nervous system, but also in the immune system [276], tumor cells [277], and lungs [277]. The latter finding is pivotal because it opens the chance of being therapeutically useful to limit the symptoms of acute and chronic lung diseases [277]. In support of this, high-altitude patients with higher EPO levels and higher hematocrits during COVID had a better chance of survival in the intensive care units [278]. Remarkably, EPO also protects the endothelium and excessive inflammation of vascular beds [279].

An additional link between EPO and metabolism comes from the observation that, in an in vitro model of Parkinson’s disease characterized by augmented redox imbalance [280], EPO accelerates the glycolytic rate without affecting the mitochondrial function, contributing to restoring in part the ATP levels in a manner similar to hypoxia adaptation [281]. This finding, however, requires in vivo validation, as EPO typically reaches its peak a few days after the onset of hypoxia and gradually returns to baseline over the following weeks [282]. EPO is not the sole factor involved in this response; its effects are mediated by binding to EPO receptors on the surface of target cells—primarily in the bone marrow and nervous system—which subsequently activate an array of intracellular signaling cascades [283]. In parallel, a soluble form of the EPO receptor, which acts as an endogenous antagonist by competing with membrane-bound EPO receptor, has been shown to decrease and remain below baseline levels for at least 72 h in humans exposed to 12% O_2_, with a slow return to baseline thereafter [284]. The role of the soluble form of the EPO receptor must be tested not only for the potentially protective effects of EPO in clinical and AIPH, but also against the classical observations that Tibetans do not display marked erythrocytosis [285], and that their plasma EPO levels are essentially the same in high- and low-altitude Tibetans [286]. By contrast, Andean altitude dwellers display excessive erythrocytosis [287], which is believed to foreplay the onset of CMS. This concept is challenged by a recent publication on CMS from the point of view of high-altitude physicians, where the increase in Red Blood Cells/Hematocrit/Hb is defined as polyerythrocytemia resulting from multiple diseases and not from mere exposure to hypobaric hypoxia. CMS is defined as an adaptative process of hypoxicating diseases at high altitude. Furthermore, EPO plays a fundamental role in the protection of vascular endothelium in high altitude residents, implying its possible favorable role in PH. It could not only reduce thromboembolic events but also reduce endothelial inflammation probable precursors of PH [7]. Remarkably, although higher EPO level would be expected, South American dwellers at 4340 m/12.0% O_2_, circulating EPO cannot be used to discriminate for CMS, in the favor of the EPO-to-EPO receptor ratio [288]. Likewise, the EPO level was only marginally higher in Andean highlanders with high and low Hb [289].

## 4. Conclusions

The molecular mechanisms linking hypoxia to PH reveal an intricate signaling network encompassing redox imbalance, the PI3K–Akt pathway, Na^+^/H^+^ exchange, nitric oxide signaling, autophagy, mitochondrial dynamics, mitophagy, metabolic reprogramming, inflammation, adventitia, and PAAF, as well as erythropoietin. Although each of these pathways exerts distinct and pivotal effects, their tight interconnection highlights the multifactorial nature of the hypoxia–PH axis. Advancing our understanding of how these components interact will be essential to identify novel therapeutic targets and to develop more effective strategies against this devastating disease.

## Figures and Tables

**Table 1:** List and definition of pulmonary hypertension (PH) as approved by the World Health Organization.

WHO Group	Name	Definition
1	Pulmonary arterial hypertension (PAH)	Idiopathic, heritable, drug-induced, associated with conditions like connective tissue diseases, human immunodeficiency viruses, congenital heart disease, and portal hypertension.
2	PH due to left heart disease	Caused by systolic/diastolic dysfunction or valvular disease affecting left heart pressures.
3	PH due to lung diseases and/or hypoxia	Linked to chronic obstructive pulmonary disease (COPD), interstitial lung disease, sleep apnea, and chronic high-altitude exposure.
4	Chronic thromboembolic PH	Caused by unresolved pulmonary emboli leading to obstructed blood flow.
5	PH with unclear or multifactorial mechanisms	Hematologic disorders, systemic diseases (e.g., sarcoidosis), metabolic disorders, and others with complex or poorly understood causes.

## Data Availability

No new data were created or analyzed in this study.

## Abbreviations

ADP	adenosine diphosphate
AIPH	altitude-induced PH
Akt	protein kinase b
AMP	adenosine monophosphate
AMPK	adenosine monophosphate kinase
ARDS	acute respiratory distress syndrome
ATP	adenosine triphosphate
cGMP	cyclic guanosine monophosphate
CMS	chronic mountain sickness
COPD	chronic obstructive pulmonary disease
eNOS	endothelial NO synthase
EPAS-1	endothelial PAS domain protein 1 or HIF-2α
EPO	erythropoietin
FUNDC1	FUN 14 domain-containing 1
Hb	hemoglobin
HIF	hypoxia-inducible factor
HPV	hypoxic pulmonary vasoconstriction
IFN-γ	interferon-γ
IL	interleukins
L-Arg	L-arginine
LC3-II	protein light chain 3 type II
LV	left ventricle
MAPK	mitogen-activated protein kinases
MnSOD	manganese superoxide dismutase
mPAP	mean pulmonary arterial pressure
NF-κB	nuclear factor-κB
NHE	Na+/H+ exchange
NOX	NADPH oxidase
Nrf-2	nuclear factor erythroid-related factor 2
P62	sequestosome
PAAF	pulmonary arterial adventitial fibroblasts
PAEC	pulmonary artery endothelial cell
PAH	pulmonary artery hypertension
PASMC	pulmonary artery smooth muscle cell
PDE	phosphodiesterase
PGC-1α	peroxisome proliferator-activated receptor gamma co-activator-1α
PH	pulmonary hypertension
PHD	prolyl hydroxylase
pHi	intracellular pH
PI3K	phosphoinositide 3-kinases
Pink1	PTEN-induced kinase 1
PKCβ	protein kinase C beta type
PKG	protein kinase G
PPARy	peroxisome proliferator-activated receptor gamma
RNS	reactive nitrogen species
ROS	reactive O2 species
RV	right ventricle
SMYD2	SET and MYND domain-containing 2
SuHx	Sugen-hypoxia
TGFβ	transforming growth factors beta
TNFα	tumor necrosis factor-α
TRPV4	transient receptor potential cation channel subfamily V member 4
UCP2	uncoupling protein 2
VEGF	vascular endothelial growth factor
VEGFR2	vascular endothelial growth factor receptor 2
